# Carisoprodol-induced amnestic state

**DOI:** 10.4103/0019-5545.39769

**Published:** 2008

**Authors:** Arun Gupta, K. Sreejayan, Prabhat Chand, Vivek Benegal, Pratima Murthy

**Affiliations:** Department of Psychiatry, National Institute of Mental Health and Neurosciences, Bangalore, Karnataka, India E-mail: chand@nimhans.kar.nic.in

Sir,

A 35-year-old man was admitted with a complaint of abuse of carisoprodol. The patient had history of opioid dependence but currently abstinence since last 2 years. After stopping opioid use, he started using carisoprodol tablets, initially 700 mg/day, increasing over a period of 2 years to 1,050 mg/day. When he presented to our center, he satisfied the criteria for dependence (ICD 10),[1] with prominent tolerance, craving, and salience. Following carisoprodol use, he described 10-15 episodes, during which he had walked for 3-4 km without any memory of events that had occurred on the way, and he could not remember the events when other people narrated those to him. However, he would not lose way and would reach home every time during these episodes. He would not identify people whom he had known before, when he met them on the way during these periods. Each of these episodes would last from 45 min to 1 h. The patient had experienced his last episode 1 week before he presented to us.

He did not report recent use of alcohol or other drugs, symptoms of aura or postictal state, recent head injury, or any other illness. The last intake of tablets was 2 days prior to admission. He did not manifest significant withdrawal symptoms except for mild body ache. Physical examination showed vital signs within normal limits. He was alert, oriented; and he scored 30 out of 30 on the mini mental state examination (MMSE).[2] Electrolytes, liver function tests, complete blood counts were within normal limits. Urine screening for cannabis, opioids, benzodiazepines, amphetamines, and cocaine were negative. His EEG (electroencephalogram) was normal.

Carisoprodol, a synthetic congener of meprobamate, is a centrally acting muscle relaxant indicated in acute painful musculoskeletal conditions.[3] An extensive literature search did not reveal any prior reports of amnestic states with carisoprodol alone. There is a report of amnestic periods in a person using a combination of carisoprodol and treatment with multiple psychotropic drugs.[4] In this report, the authors reported a case of opioid dependence, seizure disorder with major depressive disorder being prescribed carisoprodol for back and neck pain. He later developed tolerance and withdrawal symptoms for carisoprodol. He was found to have amnestic episodes, which were attributed to use of multiple psychoactive medications (sertraline, zolpidem, quitiapine, gabapentin), apart from carisoprodol.[4] Recently, carisoprodol use among drivers in Norway was found to result in significant impairment and risk for accidents, irrespective of blood meprobamate concentration.[5] In the index case, our patient was not using any other drugs or medications during the period of carisoprodol use. Hence this symptom is likely to be related to carisoprodol.

There have been previous reports of carisoprodol dependence.[4,6,7] Most patients reported using carisoprodol along with opioids to decrease the withdrawal symptoms of opioids and to avoid subsequently becoming dependent on it. However, this patient had started using carisoprodol after stopping opioids. In a recent report, specific withdrawal symptoms like anxiety, tremulousness, insomnia, jitteriness, muscle twitching, and hallucinations were described. These symptoms are most likely caused by withdrawal from the meprobamate that accumulates after large amounts of carisoprodol are ingested.[8]

Carisoprodol acts by releasing its metabolite meprobamate.[3] There are reports which attest to the dependence-causing potential of meprobamate. Though there is a report of memory deficits as assessed on neuropsychological tests in a 53-year-old man on diazepam and meprobamate,[5] a literature search did not reveal amnestic episodes with meprobamate. The exact mechanism of carisoprodol in the central nervous system is not known; it is assumed that it acts through GABA_A_ receptor. There is a case report where benzodiazepine antagonist flumazenil was used to reverse a case of carisoprodol intoxication.[10] This supports that carisoprodol may be a GABA_A_ receptor indirect agonist with central nervous system chloride ion channel conductance effects similar to the benzodiazepines.

**Organization:** National Institute of Mental Health and Neurosciences; Place: Bangalore; Date: 02-08-2007
